# Thousands of protein linear motif classes may still be undiscovered

**DOI:** 10.1371/journal.pone.0248841

**Published:** 2021-05-03

**Authors:** Denys Bulavka, Ariel A. Aptekmann, Nicolás A. Méndez, Teresa Krick, Ignacio E. Sánchez

**Affiliations:** 1 Laboratorio de Fisiología de Proteínas, Facultad de Ciencias Exactas y Naturales, Consejo Nacional de lnvestigaciones Cientificas y Técnicas, Instituto de Química Biológica de la Facultad de Ciencias Exactas y Naturales (IQUIBICEN), Universidad de Buenos Aires, Buenos Aires, Argentina; 2 Departamento de Matematica, Facultad de Ciencias Exactas y Naturales, Universidad de Buenos Aires, Buenos Aires, Argentina; 3 Department of Marine and Coastal Sciences, Rutgers University, New Brunswick, NJ, United States of America; 4 Departamento de Matematica, Facultad de Ciencias Exactas y Naturales and IMAS—CONICET, Universidad de Buenos Aires, Buenos Aires, Argentina; Centro de Investigaciones Biologicas, SPAIN

## Abstract

Linear motifs are short protein subsequences that mediate protein interactions. Hundreds of motif classes including thousands of motif instances are known. Our theory estimates how many motif classes remain undiscovered. As commonly done, we describe motif classes as regular expressions specifying motif length and the allowed amino acids at each motif position. We measure motif specificity for a pair of motif classes by quantifying how many motif-discriminating positions prevent a protein subsequence from matching the two classes at once. We derive theorems for the maximal number of motif classes that can simultaneously maintain a certain number of motif-discriminating positions between all pairs of classes in the motif universe, for a given amino acid alphabet. We also calculate the fraction of all protein subsequences that would belong to a motif class if all potential motif classes came into existence. Naturally occurring pairs of motif classes present most often a single motif-discriminating position. This mild specificity maximizes the potential number of coexisting motif classes, the expansion of the motif universe due to amino acid modifications and the fraction of amino acid sequences that code for a motif instance. As a result, thousands of linear motif classes may remain undiscovered.

## 1 Introduction

Natural proteins are synthesized as linear polymers from an alphabet of twenty amino acids, which may later be expanded through post-translational modifications. The proteome is the entire set of proteins that is, or potentially could be, expressed by an organism. Proteins present remarkable physicochemical properties that are strongly linked to the biological processes they partake in and can, in some cases, be assigned to a defined region of its sequence, such as for enzyme catalysis or folding into globular domains.

**Fig 1:**
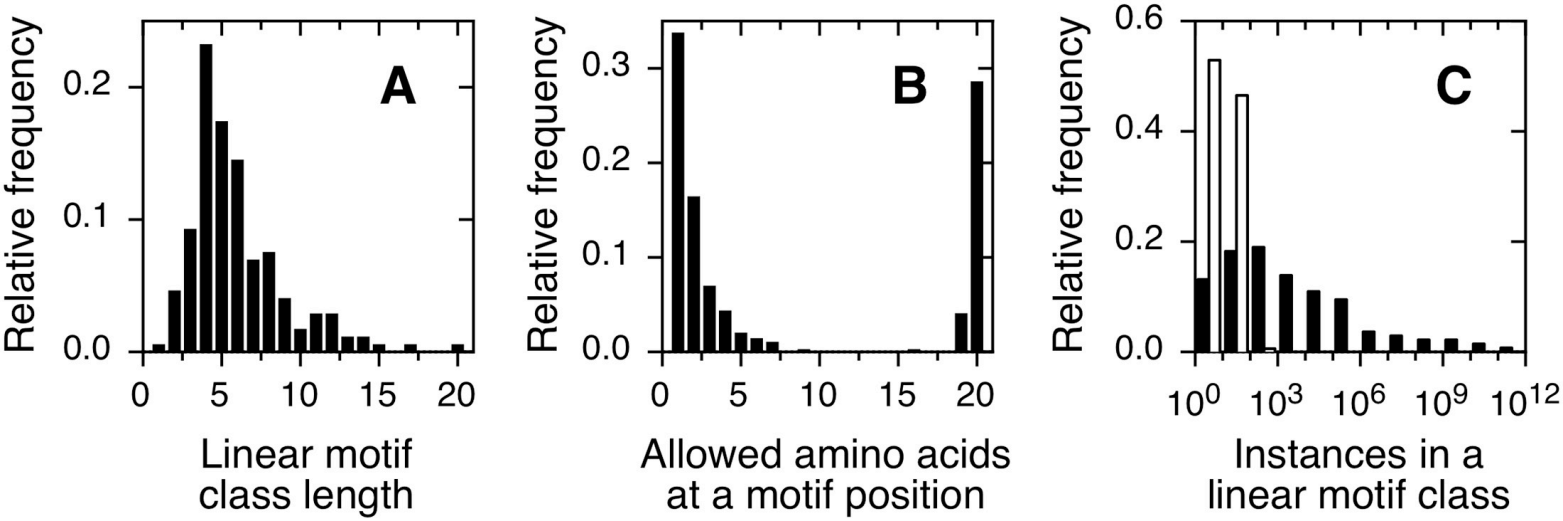
Characteristics of linear motif classes in the ELM database. (A) Histogram of observed linear motif class lengths. The total number of classes is 172. (B) Histogram of allowed amino acids (*e*_*i*_) at each motif position. The total number of positions is 1028. (C) Histogram for the number of instances within a linear motif class. Empty bars: known instances from the ELM database. Black bars: potential unique instances calculated from the corresponding regular expression. The total number of classes is 172.

**Fig 2:**
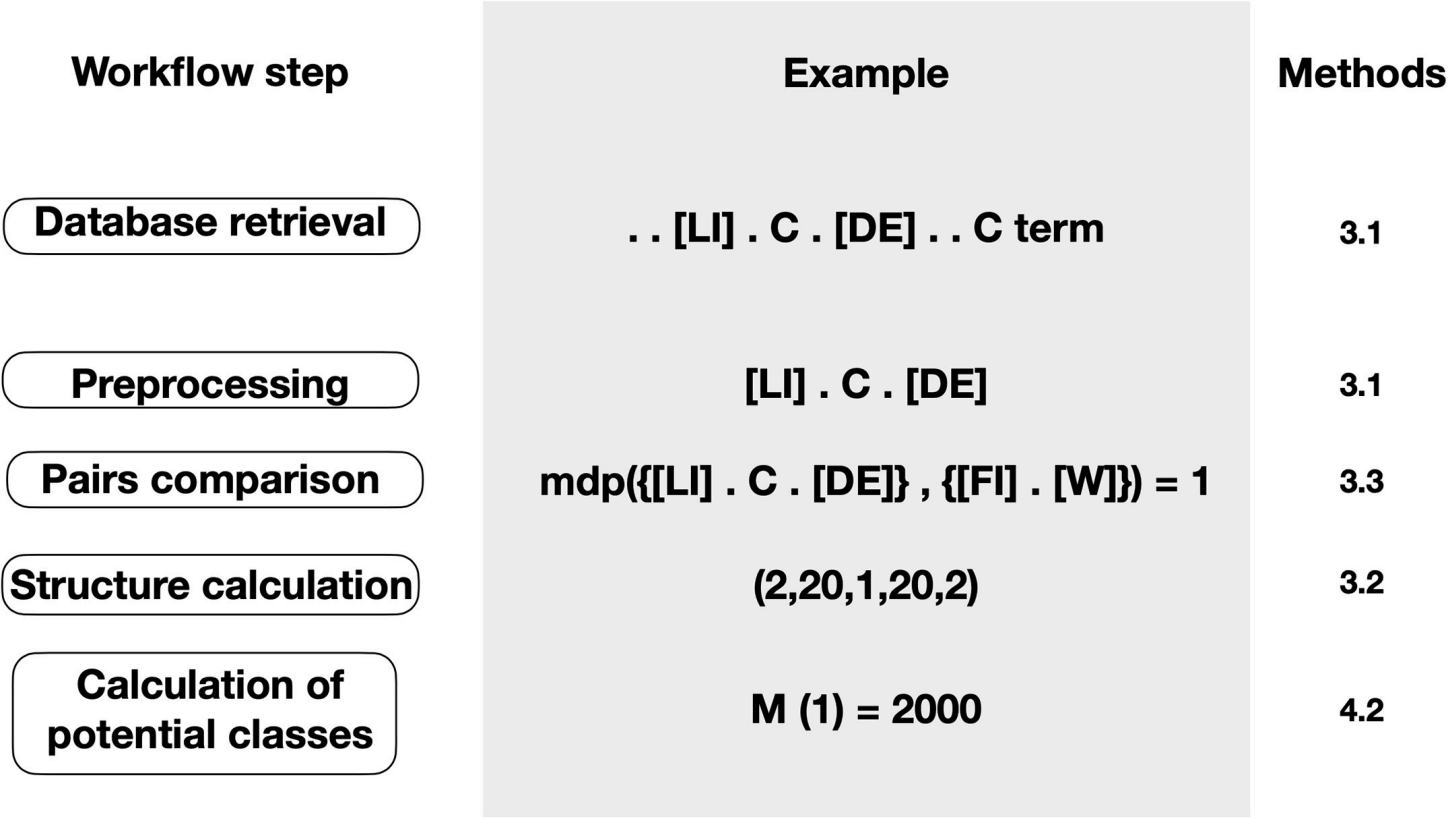
Workflow description. Process from the raw data for motif classes found in ELM db to the calculation of the potential number of motif classes.

Linear motifs, also called short linear motifs (SLiMs) or eukaryotic linear motifs (ELMs) are contiguous protein subsequences that mediate a significant fraction of protein-protein interactions in eukaryotic organisms [1]. These protein-protein interactions take place between the linear motifs and specific protein globular domains [1]. Linear motifs are usually less than 15 residues long (Fig 1A, [2]) and reside within intrinsically disordered regions that do not present a stably folded structure [2]. Linear motifs often signal post-translational modification sites or depend on post-translational modification to be active [1–3]. Since linear motifs can appear or disappear with a small number of mutations, they play an important role in the evolution of protein-protein interaction networks [1, 2, 4], particularly in metazoa [5].

A linear motif instance is a subsequence in a particular protein that is reported to perform a function by experimental and/or computational methods [3]. For example, the LYCYE subsequence in the human papillomavirus type 16 E7 protein is known to mediate binding to the human retinoblastoma protein (Rb) [4]. After the discovery of multiple linear motif instances mediating interactions with the same target protein, a linear motif class may be proposed. For example, instances belonging to the *Rb pocket B binding ligand* linear motif class mediate binding to the human retinoblastoma protein [4].

The sequences of all instances within a class are usually combined with additional biochemical, structural, mutagenesis and functional information to derive a regular expression that describes which protein sequences may belong to the corresponding linear motif class [3]. For example, the regular expression for the *Rb pocket B binding ligand* linear motif class can be written as [LI].C.[DE] [4]. In other words, protein sequences starting with a leucine or an isoleucine, followed by any amino acid, followed by a cysteine, followed by any amino acid, followed by an aspartic or glutamic acid, are expected to mediate binding to the human retinoblastoma protein when placed into a suitable context. Remarkably, to a first approximation regular expressions are valid across a range of organisms [3]. In the regular expressions reported to date, we can distinguish fixed versus wildcard positions [4]. We call fixed positions those that allow for one or a few amino acids, while we call wildcard positions those that allow for all twenty amino acids or only forbid a single amino acid (Fig 1B, [2]).

The first linear motif class was proposed nearly three decades ago [6]. Although experimental discovery of linear motif instances remains a time-consuming and error-prone process [7], close to 300 linear motif classes including over 3500 instances have been described since [3]. The number of known instances per linear motif class is shown in Fig 1C, empty bars. The class discovery rate has remained roughly unchanged for the last decade [3], suggesting that the current discovery methods are far from reaching saturation and that the undiscovered linear motif classes outnumber the known ones [8]. This situation calls for a theoretical estimation of the size and granularity of the linear motif universe. Previous work on this question has used prediction methods independent from regular expressions to estimate the number of linear motif instances in the human proteome, with a resulting figure in the range of 10^5^ to 10^6^ [9]. Although this number is helpful, the number of linear motif classes in the human proteome was not explicitly considered.

Multiple factors may impose limits on the number of linear motif classes. In this work, we focused on the limits imposed by sequence specificity. The functionality of a linear motif can be modulated in a physiological state-dependent manner to induce a gain, loss, or exchange of binding partners, which will affect the function of the protein. As such, these conditional interactions underlie molecular decision-making in cell signaling. This postulate implies that motifs are forced to bind a limited number of domains to avoid noise in the signaling process [10]. On the other hand, most proteins that participate in cellular signalling networks contain modular protein-interaction domains. Multiple versions of such domains are present within a given organism, the yeast proteome, for example, contains 27 different Src homology 3 (SH3) domains [11]. This raises the potential problem of cross-reaction. We assume that cross-talk between linear motif classes is generally avoided in natural systems [12]. That is, natural protein subsequences that are an instance of multiple linear motif classes are rare [13] and most of them are an instance of a single linear motif class.

In this work, we use theoretical tools and an empirical analysis of regular expressions in the Eukaryotic Linear Motif database [3] to investigate the specificity of natural linear motifs, how many more linear motif classes remain to be discovered, the influence of post-translational modifications, and the consequences for protein sequence space usage.

## 2 Methods

Fig 2 gives a general description of our workflow, starting from the raw data for motif classes found in ELM db and ending in the calculation of the potential number of motif classes. The details of the methods used are explained in the following sections.

### Database of linear motif classes

We retrieved all available 210 linear motif classes and corresponding regular expressions from the ELM database in May 2015 (Fig 2). Our code and primary data are available at https://gitlab.kam.mff.cuni.cz/bulavkad/elm_processing.

The preprocessing step in Fig 2 involves simplifying the regular expressions by:

- Using the shortest version of motifs with variable length. For example:
  [*LIVMP*].0, 2(*T*)*P*‥([*ST*]) to [*LIVMP*](*T*)*P*‥([*ST*])
- Not including in the regular expression N- or C-terminus. For example:
  ^*M*0,1([*ED*]) to *M*0, 1([*ED*]), and *F*‥*F*$ to *F*‥*F*
- Ignoring post-translational amino acid modifications. For example:
  RV.PU to RV.PC
- Removing flanking positions until the first and last positions allow less than eleven amino acids. For example:
  [*KR*]*R*. to [*KR*]*R*

Some linear motif classes in the ELM database correspond to minor variants of another motif and have the same biological role. This leads to regular expressions that are very similar. We inspected classes with the same name and zero discriminating positions (see below for a definition of linear motif specificity in terms of discriminating positions). In such cases, we chose to keep only the linear motif class with the highest number of instances. This led us to discard some classes, a detail of the classes we discarded and the motif representative of the group can be found in S1 Table in S1 Data.

The final number of linear motif classes and associated regular expressions in our database is 172. The complete list can be found at S1 File in S1 Data.

### Number of potential linear motif classes

We describe our calculation of the number of potential linear motif classes using regular expressions. Given the set A={A,C,D,E,F,G,H,I,K,L,M,N,P,Q,R,S,T,V,W,Y} of twenty amino acids that are used to build natural proteins and a natural number *n*, we define a *linear motif class (of length n)* as a sequence (*A*_1_, …, *A*_*n*_) where each *A*_*i*_ is a subset of A, and a *linear motif instance* of this class is a sequence (*a*_1_, …, *a*_*n*_) with *a*_*i*_ ∈ *A*_*i*_ for all *i* = 1, …, *n*. Given a linear motif class **A** = (*A*_1_, …, *A*_*n*_), by its *structure* we refer to the sequence (|*A*_1_|, …, |*A*_*n*_|), i.e. the number of residues at each position.

For example, following this convention, the regular expression [*LI*].*C*.[*DE*] corresponds to the linear motif class

$$\begin{array}{c} \left(\{LI\},A,\{C\},A,\{D,E\}\right) \end{array}$$

of length 5 and structure (2, 20, 1, 20, 2) and (*I*, *A*, *C*, *D*, *D*) is a linear motif instance of this class. Notice that several classes can have the same structure.

Here we fix *n* and a structure **e** = (*e*_1_, …, *e*_*n*_). We denote the set of all possible linear motif classes with this structure by $M_{e}$.

Given two classes $A=\left(A_{1},\ldots ,A_{n}\right),B=\left(B_{1},\ldots ,B_{n}\right)\in M_{e}$, we say that *they have (at least) one motif-discriminating position* if there is at least one coordinate *ℓ*, 1 ≤ *ℓ* ≤ *n*, such that *A*_*ℓ*_ ∩ *B*_*ℓ*_ = ∅. This corresponds to the fact that these two classes are indeed biologically different linear motif classes, since they cannot share any linear motif instance.

For example, for **e** = (7, 6) the two classes ({*A*, *C*, *D*, *E*, *F*, *G*, *H*}, {*A*, *C*, *D*, *E*, *F*, *G*}) and ({*A*, *C*, *D*, *E*, *F*, *G*, *H*}, {*H*, *I*, *K*, *L*, *M*, *N*}) have one motif-discriminating position since looking at their second coordinate we observe that {*A*, *C*, *D*, *E*, *F*, *G*}∩{*H*, *I*, *K*, *L*, *M*, *N*} = ∅.

Given a set of linear motif classes $\left\{A^{\left(1\right)},\ldots ,A^{\left(m\right)}\right\}\subset M_{e}$, we say that it is 1-discriminating if any two different classes in it have at least one motif-discriminating position, that is for any *i* ≠ *j*, **A**^(*i*)^ and **A**^(*j*)^ have at least one coordinate *ℓ* such that $A_{\ell }^{\left(i\right)}\cap A_{\ell }^{\left(j\right)}=\emptyset$. This corresponds to the fact that all classes in this set are indeed biologically different linear motif classes. We are interested in the maximal possible size (number of elements) of such a 1-discriminating set in $M_{e}$, since it provides us information on the maximal possible number of different biological linear motif classes of given structure **e**. This is the first problem we pose.

**Problem 1**. *Given the structure* **e** = (*e*_1_, …, *e*_*n*_), *how big can a 1-discriminating set in* $M_{e}$ *be?*

*Remark*. There exists a 1-discriminating set in $M_{e}$ of size $\geq \prod_{1\leq i\leq n}\left\lfloor \frac{20}{e_{i}}\right\rfloor$. (Here, for a positive real number *r*, ⌊*r*⌋ denotes its *floor*, the maximal integer number *k* with *k* ≤ *r*).

*Proof*. For each *i* we build $p_{i}≔\left\lfloor \frac{20}{e_{i}}\right\rfloor$ pairwise disjoint subsets $A_{i}^{\left(1\right)},\ldots ,A_{i}^{\left(p_{i}\right)}$ of our set of amino acids $A=\{a_{1},\ldots ,a_{20}\}$ with *e*_*i*_ elements each: for example, we can define $A_{i}^{\left(1\right)}≔\left\{a_{1},\ldots ,a_{e_{i}}\right\}$, $A_{i}^{\left(2\right)}≔\left\{a_{e_{i}+1},\ldots ,a_{2e_{i}}\right\},\ldots ,A_{i}^{\left(p_{i}\right)}=\left\{a_{\left(p_{i}-1\right)e_{i}+1},\ldots ,a_{p_{i}e_{i}}\right\}$, where we note that *p*_*i*_ *e*_*i*_ ≤ 20 because $p_{i}=\left\lfloor \frac{20}{e_{i}}\right\rfloor \leq \frac{20}{e_{i}}$. Now we define the set consisting of all possible different sequences (*A*_1_, …, *A*_*n*_) where each of the *A*_*i*_’s is chosen among $A_{i}^{\left(1\right)},\ldots ,A_{i}^{\left(p_{i}\right)}$, 1 ≤ *i* ≤ *n*. Two such sequences have at least one different coordinate, that by construction do not intersect, so the set of all such possible sequences is 1-discriminating. We conclude by noting that there are *p*_1_⋯*p*_*n*_ such sequences.

We give an example to clarify how the proof works.

**Example 1.1**. *For* **e** = (7, 6), *we can take* $A_{1}^{\left(1\right)}=\left\{A,C,D,E,F,G,H\right\}$, $A_{1}^{\left(2\right)}=\left\{I,K,L,M,N,P,Q\right\}$, $A_{2}^{\left(1\right)}=\left\{A,C,D,E,F,G\right\}$, $A_{2}^{\left(2\right)}=\left\{H,I,K,L,M,N\right\}$, $A_{2}^{\left(3\right)}=\left\{P,Q,R,S,T,V\right\}$, *and we obtain the following set of linear motif discriminating classes in* $M_{\left(7,6\right)}$ *that has 6 elements*:

$$\begin{array}{c} \{\left(A_{1}^{\left(1\right)},A_{2}^{\left(1\right)}\right),\left(A_{1}^{\left(1\right)},A_{2}^{\left(2\right)}\right),\left(A_{1}^{\left(1\right)},A_{2}^{\left(3\right)}\right),\left(A_{1}^{\left(2\right)},A_{2}^{\left(1\right)}\right),\left(A_{1}^{\left(2\right)},A_{2}^{\left(2\right)}\right),\left(A_{1}^{\left(2\right)},A_{2}^{\left(3\right)}\right)\}. \end{array}$$

*This is a 1-discriminating set in* $M_{\left(7,6\right)}$.

Next result shows that the size of all 1-discriminating sets in $M_{e}$ is bounded by a quantity which is roughly similar to the bound of Remark 1.

**Proposition 1**. *All 1-discriminating sets in* $M_{e}$ *have size* $\leq \prod_{1\leq i<n}\frac{20}{e_{i}}$.

In order to prove this proposition we need the following lemma.

**Lemma 1.2**. *Let e be a natural number and* $A_{1},\ldots ,A_{m}\subseteq A$ *with* |*A*_*i*_| = *e*, *i* = 1, …, *m*. *Set* $k≔\lceil \frac{me}{20}\rceil$ *(here, for a positive real number r*, ⌈*r*⌉ *denotes its ceiling*, *the minimal integer number k with k* ≥ *r*). *Then there exists an amino acid* a∈A *such that a belongs to at least k of the sets A*_1_, …, *A*_*m*_, *that is, there exists* {*i*_1_, …, *i*_*k*_} ⊂ {1, …, *m*} *such that* $a\in A_{i_{j}}$ *for* 1 ≤ *j* ≤ *k*.

*Proof*. This is a consequence of the famous pigeonhole principle which says that if in a pigeon loft there are more pigeons than holes, then there are at least to pigeons in the same hole. Note that if *r* is an integer number, then ⌊*r*⌋ = *r* = ⌈*r*⌉ while if *r* is a non-integer positive real number, then ⌊*r*⌋ < *r* < ⌈*r*⌉ and ⌈*r*⌉ = ⌊*r*⌋ + 1.

Let us denote $A=\{a_{1},\ldots ,a_{20}\}$. The disjoint union of the *A*_*i*_ (counting each element one time as if they were all different) has *me* elements. We start with the simpler case when $\frac{me}{20}$ is an integer number, i.e. $k=\frac{me}{20}$: If for 1 ≤ *i* ≤ 20, each $a_{i}\in A$ belongs to *k*_*i*_ < *k* of the sets *A*_1_, …, *A*_*m*_, then one would have *me* = *k*_1_ + ⋯ + *k*_20_ < 20 ⋅ *k* = *me*, a contradiction. Now let us consider the case when $\frac{me}{20}$ is not an integer number: Again, if for 1 ≤ *i* ≤ 20, each $a_{i}\in A$ belongs to $k_{i}<k=\left\lceil \frac{me}{20}\right\rceil$, that is to $k_{i}\leq \left\lfloor \frac{me}{20}\right\rfloor$, of the sets *A*_1_, …, *A*_*m*_, then one would have

$$\begin{array}{c} me=k_{1}+\cdots +k_{20}\leq 20\cdot \left\lfloor \frac{me}{20}\right\rfloor <20\cdot \frac{me}{20}=me, \end{array}$$

again a contradiction. Thus, in both cases there exists at least one a∈A which belongs to at least *k* of the sets *A*_1_, …, *A*_*m*_.

*Proof of Proposition 1*. Let ({**A**^(1)^, …, **A**^(*m*)^} be such a 1-discriminating set, with $A^{\left(i\right)}=\left(A_{1}^{\left(i\right)},\ldots ,A_{n}^{\left(i\right)}\right)$ for *i* = 1, …, *m*. Since $A_{1}^{\left(1\right)},\ldots ,A_{1}^{\left(m\right)}\subseteq A$ with $|A_{1}^{\left(i\right)}|=e_{1}$ for *i* = 1, …, *m*, by Lemma 1.2, there exists $a_{1}\in A$ which belongs to at least $\left\lceil \frac{me_{1}}{20}\right\rceil$ sets $A_{1}^{\left(i\right)}$. Now consider all these *i* such that $a_{1}\in A_{1}^{\left(i\right)}$, repeating the reasoning for $A_{2}^{\left(i\right)}$, by Lemma 1.2, there exists $a_{2}\in A$ which belongs to at least $\left\lceil \frac{\left\lceil \frac{me_{1}}{20}\right\rceil e_{2}}{20}\right\rceil$ of these $A_{2}^{\left(i\right)}$. Iterating, it follows that there is an element $a_{n-1}\in A$ that belongs to

$$\begin{array}{c} k≔\lceil \frac{\lceil \cdots \lceil \frac{me_{1}}{20}\rceil \cdots \rceil e_{n-1}}{20}\rceil \end{array}$$

of the $A_{n-1}^{\left(i\right)}$. This implies that all $A_{j}^{\left(i\right)}$ intersect for fixed *j*, 1 ≤ *j* ≤ *n* − 1. Since these **A**^(*i*)^ form a 1-discriminating set, it must happen that for any two indexes *i*_1_ and *i*_2_, $A_{n}^{\left(i_{1}\right)}\cap A_{n}^{\left(i_{2}\right)}=\emptyset$. This implies *ke*_*n*_ ≤ 20. Since it can be shown recursively that

$$\begin{array}{c} m\prod_{1\leq i\leq n-1}\frac{e_{i}}{20}\leq \lceil \frac{\lceil \cdots \lceil \frac{me_{1}}{20}\rceil \cdots \rceil e_{n-1}}{20}\rceil =k, \end{array}$$

one also has

$$\begin{array}{c} me_{n}\prod_{1\leq i\leq n-1}\frac{e_{i}}{20}=ke_{n}\leq 20, \end{array}$$

and we conclude that $m\leq \prod_{1\leq i\leq n}\frac{20}{e_{i}}$.

**Example 1.3**. *For* **e** = (7, 6), *the previous result shows that any* 1-*discriminating set has at most* $\frac{20}{7}\cdot \frac{20}{6}=11,11...$, *that is at most 11 linear motif classes in it, and we already know there exists at least one 1-discriminating set with 6 classes in it. Notice that in this case the proof itself yields the more precise bound 7, instead of 11. As another example, for* **e** = (4, 5), *Remark 1 shows that there exists a 1-discriminating set of size* $\geq \left\lfloor \frac{20}{4}\right\rfloor \cdot \left\lfloor \frac{20}{5}\right\rfloor =20$ *while by Proposition 1, all 1-discriminating sets have size* $\leq \frac{20}{4}\cdot \frac{20}{5}=20$ *as well, so the lower and upper bounds match in this case*.

One can wonder why it seems experimentally that nature selects linear motif classes to be distinct when they have at least one discriminating position when considered two by two. This is somehow justified by the numbers we analyze in the sequel, that show that 1-discriminating sets give many more possibilities than any other selection nature could have made. For this purpose we introduce the next definitions which generalize the definition of 1-discriminating set.

Given two classes $A=\left(A_{1},\ldots ,A_{n}\right),B=\left(B_{1},\ldots ,B_{n}\right)\in M_{e}$ and *k* ≥ 1, we say that *they have at least k motif-discriminating positions* if there are at least *k* coordinates *j*, 1 ≤ *j* ≤ *n* such that *A*_*j*_ ∩ *B*_*j*_ = ∅. And given a set of linear motif classes $\left\{A^{\left(1\right)},\ldots ,A^{\left(m\right)}\right\}\subset M_{e}$, we say that it is *k-discriminating* if any two different classes in it have at least *k* motif-discriminating positions. This leads us to our second problem.

**Problem 2**. *Given the structure* **e** = (*e*_1_, …, *e*_*n*_), *how big can a k-discriminating set in* $M_{e}$ *be?*

Our answer, which is proven as Proposition 1, is as follows.

**Proposition 2**. Let *k* ≥ 1. All *k*-discriminating sets in $M_{e}$ have size $\leq \prod_{1\leq i\leq n-(k-1)}\frac{20}{e_{i}}$.

Note that when applying this proposition, we can choose to order the *e*_*i*_s from larger to smaller, so that the obtained upper bound is sharper.

We finally define and study the concept of 0-discriminating set. Given two classes $A=\left(A_{1},\ldots ,A_{n}\right),B=\left(B_{1},\ldots ,B_{n}\right)\in M_{e}$, we say that *they present 0 motif-discriminating positions* when *A*_*i*_ ∩ *B*_*i*_ ≠ ∅ for all 1 ≤ *i* ≤ *n*. Accordingly, given a set of linear motif classes $\left\{A^{\left(1\right)},\ldots ,A^{\left(m\right)}\right\}\subset M_{e}$, we say that it is *0-discriminating* if any two different classes in it pres-ent 0-discriminating positions. This problem is

**Problem 3**. *Given the structure* **e** = (*e*_1_, …, *e*_*n*_), *how big can a 0-discriminating set in* $M_{e}$ *be?*

The problem is of major interest when *e*_*i*_ ≤ 10, 1 ≤ *i* ≤ *n*, since if not all subsets in each coordinate where *e*_*i*_ > 10 intersect 2 by 2.

**Proposition 3**. *Let* **e** = (*e*_1_, …, *e*_*n*_) *with e*_*i*_ ≤ 10 *for* 1 ≤ *i* ≤ *n*. *Then all 0-discriminating sets in* $M_{e}$ have size ≤∏1≤i≤n(19ei−1).

*Proof of Proposition 3*. The famous Erdös-Ko-Rado theorem in Combinatorics gives us exactly the answer for *n* = 1: It says that if one has *m* subsets of A of size *e*_1_, with 20 ≥ 2*e*_1_, such that each pair of subsets has a non-empty intersection, then m≤(19e1−1). For the general case *n* ≥ 2, this is the cardinal of a Cartesian product: In the first coordinate, we can choose at most k1=(19e1−1) sets, in the second coordinate, we can choose at most k2=(19e2−1) sets and in the *n*-th coordinate, we can choose at most kn=(19en−1). The conclusion follows.

### Sequence specificity of linear motif classes

In practice we want to ensure that only fixed positions of the regular expression are taken into account, this motivates the following definition of specificity; given two linear motif classes with the same length *n*, but possibly different structure, **A** = (*A*_1_, …, *A*_*n*_) and **B** = (*B*_1_, …, *B*_*n*_), by *motif-discriminating positions* we refer to the number of fixed positions *with at most 10 allowed residues* where no amino acid can match both regular expressions, i.e.

$$\begin{array}{c} mdpAB=|\{i\in \left\{1,\cdots ,n\right\}:A_{i}\cap B_{i}=\emptyset \;\text{with}\;|A_{i}|\leq 10\;\text{and}\;|B_{i}|\leq 10\}|. \end{array}$$ (1)

For example, for the regular expressions [*LI*].*C* and [*FI*].*W* represented by the linear motif clas-ses A=({LI},A,{C}) and B=({FI},A,{W}), one has *mdp* **AB** = 1 corresponding to the fact that *A*_3_ ∩ *B*_3_ = ∅ with |*A*_3_| ≤ 10 and |*B*_3_| ≤ 10.

Given the same linear motifs **A** and **B**, we indicate that we only care about what happens at matching positions with at most 10 residues by introducing the following notation.

$$\begin{array}{c} alignvalAB=\{\begin{array}{cc} 1 & \text{if}\;\text{there}\;\text{exists}\;i,\;1\leq i\leq n,\;\text{such}\;\text{that}\;|A_{i}\left|\leq 10\;\text{and}\;\right|B_{i}|\leq 10,\\ 0 & \text{otherwise.}\; \end{array} \end{array}$$

For the previous example we note that *alignval* **AB** = 1.

The rest of this subsection is concerned with extending the definition of motif-discriminating positions to compare classes of different lengths. Given two linear motifs of different lengths **A** = (*A*_1_, …, *A*_*n*_) and **B** = (*B*_1_, …, *B*_*m*_), with *n* ≥ *m*, we can define a *set of alignments* between them as

$$\begin{array}{c} alignsetAB=\{((A_{1},\cdots ,A_{m}),B),((A_{2},\cdots ,A_{m+1}),B),\cdots ,((A_{n-m+1},\cdots ,A_{n}),B)\}. \end{array}$$ (2)

For example, for the linear motif classes A=({LI},A,{C},A,{DE}) and B=({FI},A,{W}) the set of alignments has 3 elements, namely

$$\begin{array}{c} alignsetAB=\{((\{LI\},A,\{C\}),(\{FI\},A,\{W\})),\\ ((A,\{C\},A),(\{FI\},A,\{W\})),((\{C\},A,\{DE\}),(\{FI\},A,\{W\}))\}. \end{array}$$ (3)

**Fig 3:**
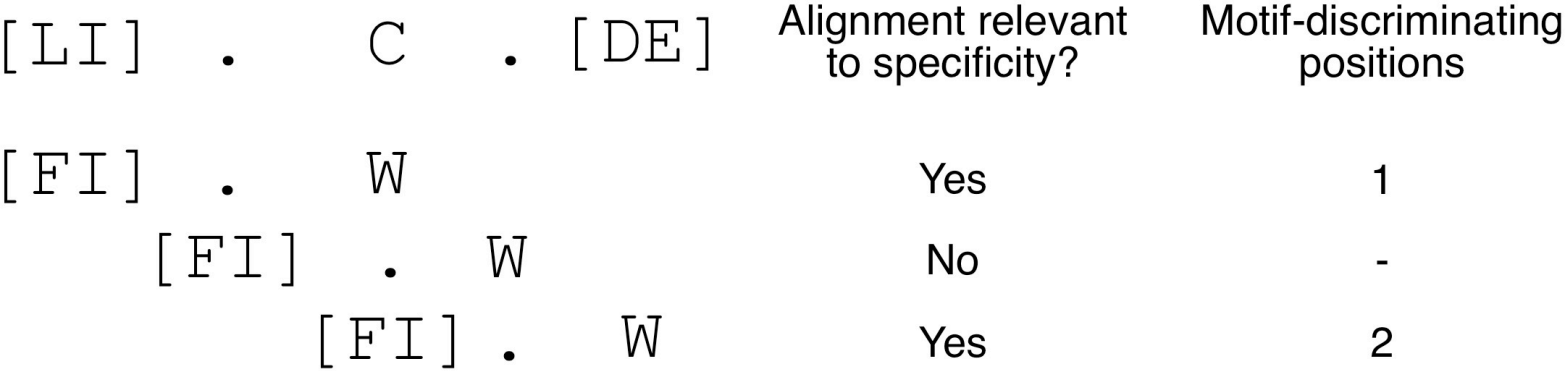
Measurement of the distance in sequence space between a pair of linear motif classes. We illustrate the calculation for the regular expressions [LI].C.[DE] and [FI].W. Due to the different lengths of the two regular expressions there are three possible alignments, all of them hanging ends that belong to the longer regular expression. The second alignment does not match a pair of fixed positions and does not help us test the distance in sequence space between the two motifs. The first and third alignments match two pairs of fixed positions each. For each of them, we count the number of motif-discriminating positions where no amino acid can match both regular expressions. The result is one for the first alignment and two for the third alignment. We take the minimum of these two figures. Thus, the distance in sequence between these two linear motif classes is of at least one motif-discriminating position.

A graphical representation of this procedure can be found in Fig 3.

In this example, *alignset* **AB** contains the 3 elements (**A**_1_, **B**), (**A**_2_, **B**) and (**A**_3_, **B**), where

$$\begin{array}{c} A_{1}=\left(\left\{LI\right\},A,\left\{C\right\}\right),\;A_{2}=(\left(A,\left\{C\right\},A\right),\;A_{3}=\left(\left\{C\right\},A,\left\{DE\right\}\right) \end{array}$$

have the same length than **B**. We note on one hand that *alignval* **A**_**1**_ **B** = *alignval* **A**_**3**_ **B** = 1 while *alignval* **A**_**2**_ **B** = 0 and on another hand that *mdp* **A**_**1**_ **B** = 1 and *mdp* **A**_**3**_ **B** = 2. This induces the definition of motif-discriminating positions for classes of possibly different lengths. Given two linear motifs **A** and **B** of lengths *n* ≥ *m*, and the corresponding set of alignments

$$\begin{array}{c} alignsetAB=\{\left(A_{1},B\right),\cdots ,\left(A_{n-m+1},B\right)\} \end{array}$$

their *motif-discriminating positions number* is defined as

$$\begin{array}{c} mdpAB=\text{min}\{mdpA_{k}B\;\text{with}\;alignvalA_{k}B=1\}. \end{array}$$ (4)

In the example we are considering, we have

$$\begin{array}{c} mdpAB=\text{min}\left\{mdpA_{1}B\right),mdpA_{1}B\}=\text{min}\left\{1,2\right\}=1. \end{array}$$

### Effective number of post-translational modifications

We identified 522 unique post-translational modifications in the Uniprot [14] ontology as of August, 2018. This number includes four Uniprot categories of modification, we present the number of occurrences of each category in S2 Table in S1 Data.

Shannon’s information theory provides a quantitative way of choosing the number of representative entities from a mixture of unevenly used symbols [15]. This method is often used, for example to determine the effective number of species in an ecosystem [16]. We first calculate the entropy *H* of the mixture as:

$$\begin{array}{c} H=\sum_{i=1}^{n}-p_{i}\;{\text{log}}_{2}\left(p_{i}\right), \end{array}$$ (5)

where *p*_*i*_ is the relative frequency of symbol *i*. According to the theory, the number of representative entities is 2^*H*^.

In the case of the mixture of 522 post-translational modifications in Swissprot the effective number of entities is 12.33, which we round to twelve. The frequency and type of these twelve post-translational modifications accounting for 87% of the Uniprot database modifications is presented on S2 Table in S1 Data.

## 3 Results

### Sequence specificity of known linear motif classes

We defined a quantitative measure of the distance in sequence space between a pair of linear motif classes (see Methods subsection 2 for details and Fig 3 for an example).

First, we consider those alignments between the two corresponding regular expressions that do not leave a hanging end for the shorter regular expression. This may underestimate the contribution of some motif flanking positions to specificity. If an alignment does not match a pair of fixed positions, we discard it because it does not help us test the distance in sequence space between the two motifs. For each of the remaining alignments, we count the number of fixed positions where no amino acid can match both regular expressions. Finally, we take the minimal number of fixed positions across all alignments. Thus, our number of motif-discriminating positions is a lower limit for the distance in sequence space between the two linear motif classes (i.e. other positions might not fully overlap). This is because two motif-determining positions that allow for multiple amino acids and only share some of them are not counted as motif-discriminating when aligned.

#### Example: [LI].C.[DE] and [FI].W

This step corresponds to the step “pairs comparison” of the workflow we present in Fig 2 and that we described on methods 1. As we show in Fig 3 there are three possible alignments between the two regular expressions [LI].C.[DE] and [FI].W. Only two of them are relevant to specificity, the one which aligns the first position of both motifs and the one which aligns the last position of both motifs. The alignment matching the second position of the [LI].C.[DE] motif and the first position of the [FI].W motif is trivial in the sense that any instance of the two motifs could match both regular expressions. The first relevant alignment has only one motif discriminating position, while the second relevant alignment has two motif-discriminating positions. Therefore, the minimal number of motif discriminating positions is one for this pair of regular expressions.

#### Global results

We considered linear motif classes reported in the ELM database (Fig 2) (see section 2 for details). We avoided redundancy by excluding linear motif classes that correspond to minor variants of another motif and have the same biological role, for a detail of the excluded motifs see S1 Table in S1 Data. This left us with 172 linear motif classes (see S1 File in S1 Data for a full list of the motifs). Our simplified approach does not take into account several features of the corresponding regular expression, such as protein termini, variable length and post-translational modifications.

**Fig 4:**
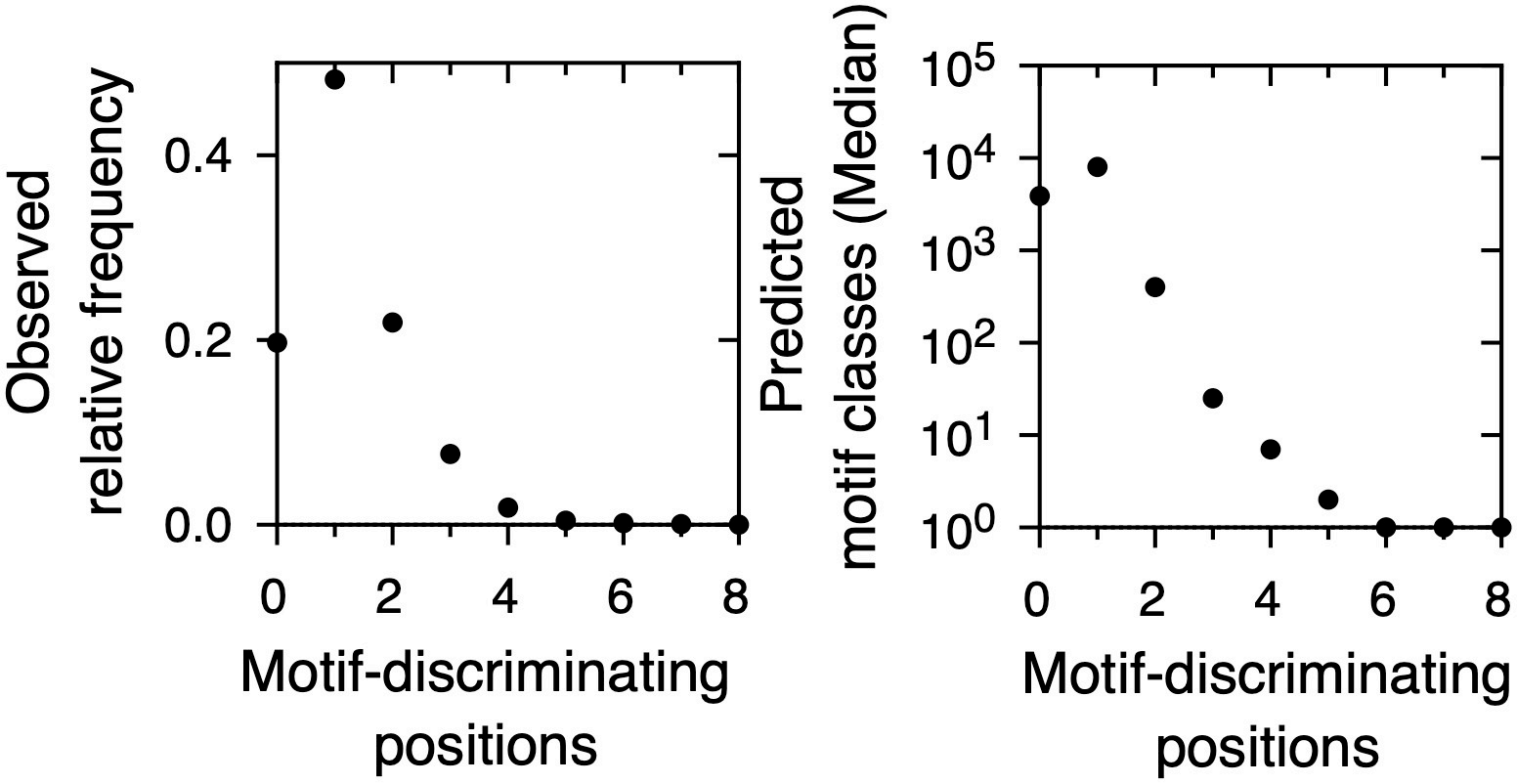
Number of potential linear motif classes as deduced from the ELM database. (Left) Number of motif-discriminating positions for all possible linear motif pairs in the database. The total number of pairs is 14706. (Right) Number of potential linear motif classes for different numbers of motif-discriminating positions.

We calculated the number of motif-discriminating positions for all possible 14706 pairs of linear motif classes in our database (Fig 4A). In about 80% of the comparisons the two regular expressions are separated in sequence space by at least one and at most eight motif-discriminating positions. The most common separation (approximately 50% of the cases) is a single motif-discriminating position, while it is rare to find regular expressions with a separation of more than three motif-discriminating positions. Out of the 20% of comparisons where the two regular expressions are not separated in sequence space by at least one motif-discriminating position, only in 3.6% of cases there is a full coincidence between the two regular expressions. We conclude that over 96% of regular expression pairs show some separation in sequence space, in agreement with our assumption that there is little crosstalk between natural linear motif classes [12] when all pairwise comparisons are taken into account. The most common value of sequence separation is a single motif-discriminating position. This is in agreement with the use of regular expressions, where a mismatch at a single position is enough to rule out that a sequence belongs to a given linear motif class.

### Number of potential linear motif classes

We used the pigeonhole principle to develop a mathematical theory that allows us to calculate the number of potential linear motif classes. This theory considers all amino acids in an alphabet as equal. However, evolutionary constraints on protein expression and the biophysics of protein interactions mediated by linear motifs may restrict the use of some amino acids and combinations thereof. In this case, the actual number of potential linear motif classes would be lower than in our model. We give the main results in this section, while the details are described in methods section 2. We consider a linear motif structure **e** = (*e*_1_, …, *e*_*n*_), where *e*_*i*_ is the number of allowed amino acids at position *i* of the regular expression. For a given structure **e** and a number *k* of motif discriminating positions, |M(k)| denotes the maximal number of linear motif classes in $M_{e}$ satisfying the property that every pair of classes in it have at least *k* motif-discriminating positions. We got the following results.

|M(0)|≤∏1≤i≤n(19ei−1), (6)

$$\begin{array}{c} \prod_{1\leq i\leq n}\left\lfloor 20/e_{i}\right\rfloor \leq \left|M\left(1\right)\right|\leq \prod_{1\leq i\leq n}20/e_{i}, \end{array}$$ (7)

$$\begin{array}{c} \left|M\left(k\right)\right|\leq \prod_{1\leq i\leq n-(k-1)}20/e_{i}\;\;\text{for}\;k<n, \end{array}$$ (8)

$$\begin{array}{c} \left|M\left(n\right)\right|=\underset{1\leq i\leq n}{\text{min}}\left\lfloor 20/e_{i}\right\rfloor . \end{array}$$ (9)

#### Example: [LI].C.[DE]

Let us perform the calculation for the regular expression [LI].C.[DE] of the *Rb pocket B binding ligand*, *LIG*_*Rb*_*LxCxE*_1 in ELM DB. Its structure is (2,20,1,20,2). If we impose that all pairs of classes present at least one motif-discriminating position, *k* = 1, the number of potential linear motif classes that can exist is (20/2) * (20/20) * (20/1) * (20/20) * (20/2) = 2000 (Table 1).

**Table 1:** The number of potential linear motif classes of the structure (2,20,1,20,2) that exist depends on the number of motif discriminating positions *k* required to differentiate two classes.

*k*	0	1	2	3	4	5
Potential classes	361	2000	100	10	1	1

We note that these figures are independent of the order of the *e*_*i*_, so that calculations for regular expressions with the structures (2,20,1,20,2) and (20,20,2,2,1) yield the same results.

#### Global results

The above equations take as input both a motif structure and a minimum number of motif-discriminating positions. We used the motif structures reported in the ELM database (Fig 1) and the numbers of motif-discriminating positions measured here (Fig 4A) to estimate the number of ELM-like linear motif classes that can potentially exist in nature. We first converted the regular expressions in our database to motif structures Fig 2. For each structure, we calculated the number of potential linear motif classes of that structure (Fig 2) for values of *k* between 0 and 8, which is the observed range of motif discriminating positions in ELM. As expected from the heterogeneity in motif lengths and structures (Fig 1), the calculated values span several orders of magnitude. In order to achieve a global view of the results, we put together the values for all regular expressions to calculate the cumulated probability that the number of potential linear motif classes is higher than a given number (S1 Fig in S1 Data). Within our highly simplified view, we decided to report the median of the distribution, i.e., the number of potential linear motif classes that has a cumulated probability of 0.5. Fig 4B shows that for *k* = 1, there is a 50% chance that the number of potential linear motif classes is at least 8000. The number of potential linear motif classes is lower at other values of *k*, taking a value of 3876 at *k* = 0 and dropping abruptly at *k* = 2 and higher. The lower value at *k* = 2 and higher is due to higher non-overlap requirements, while the lower value at *k* = 0 arises because the overlap imposed by this condition is more restrictive than the non-overlap imposed by *k* = 1. It is interesting to compare panels A and B of Fig 4. On one hand, natural linear motif pairs are most often separated in sequence space by a single motif-discriminating position. On the other hand, this relatively low level of sequence specificity maximizes the number of potential linear motif classes that can coexist while fulfilling the specificity requirement.

### Role of amino acid post-translational modifications

#### Amino acid alphabet size for protein linear motifs

The number of potential linear motif classes depends strongly on alphabet size (Eqs (6)–(9)). Traditionally, the protein alphabet is described as consisting of the 20 amino acids that are encoded by the translation machinery in all organisms. This is the number we have chosen as a basis for our calculations. However, eukaryotic organisms, where most linear motif classes have been identified, also encode for selenocysteine. Moreover, it is also well known that natural polypeptides may contain over 500 additional amino acids due to post-translational modification [17]. Thus, the number of amino acids that accurately represents the actual chemical diversity found post-translationally in proteins is higher than 21.

How many post-translational modifications should be included in an expanded, representative amino acid alphabet? Since not all 522 known post-translational modifications are present in all organisms [17] or partaking in linear motif function, we have taken two approaches to estimate the effective alphabet size in protein linear motifs. We inspected the ELM database descriptions of linear motif classes to check whether post-translational modifications are directly relevant to linear motifs in proteins. We found that multiple known motifs are dependent on the presence of at least eight modified amino acids, a summary of such motifs and residues can be found on S3 Table in S1 Data. This effectively brings the protein alphabet size up to 29. Since linear motif classes keep being discovered [3] and some of them depend on the presence of post-translational modifications, we regard 29 as a lower limit.

As a second, more general, approach we have determined an effective number of post-translational modifications in the Swissprot database using information theory (see Methods section 2 for details). In brief, we retrieved 499905 instances for all 522 post-translational modifications in Swissprot. Their relative abundances span five orders of magnitude: there are 120084 disulfide bonds in the database, while some modifications have been reported only once. Shannon’s information theory allows us to calculate the effective number of post-translational modifications in this uneven mixture as 2^*H*^, with *H* being Shannon’s entropy (See Methods section 2 for details). The result of the calculation is that the effective number of post-translational modifications is 12. This number covers over 87% of the total instances in Swissprot and includes disulfide bonds, phosphoserine, N-linked glycosylation (GlcNAc…) of asparagine, phosphothreonine, N6-acetyllysine, Glycyl lysine isopeptides, phosphotyrosine, N6-succinyllysine, N6-(pyridoxal phosphate)lysine, N-acetylalanine, S-palmytoil cysteine, and N-acetylmethionine. Taking into account selenocysteine, this generic calculation yields an effective alphabet size of 33 amino acids.

#### Example: [LI].C.[DE]

Next, we examined the effect of increasing alphabet size on the number of potential linear motif classes. As an example, we can first consider the *Rb pocket B binding ligand* linear motif class, described by the regular expression [LI].C.[DE] and the structure (2,20,1,20,2). In the case of a single motif-discriminating position, the number of potential linear motif classes is given by Eq (7) in section (1). For an alphabet size of 20, the number of potential linear motif classes of this structure is (20/2) * (20/20) * (20/1) * (20/20) * (20/10) = 2000. This number goes up to at least ⌊(29/2) * (29/29) * (29/1) * (29/29) * (29/2)⌋ = 5864 for an alphabet size of 29 and to at least ⌊(33/2) * (33/33) * (33/1) * (33/33) * (33/2)⌋ = 8448 for an alphabet size of 33.

#### Global results

**Fig 5:**
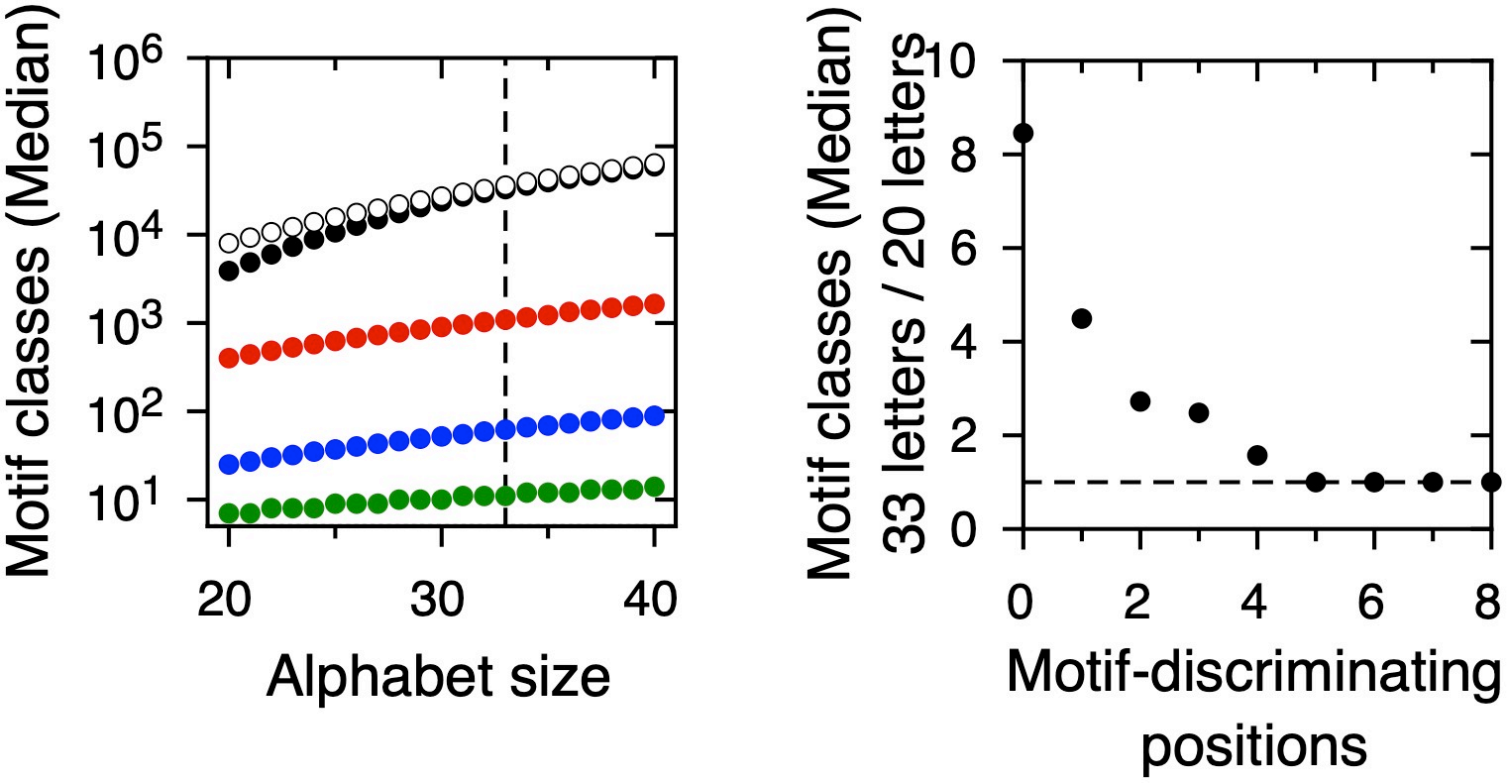
Number of potential linear motif classes as a function of protein alphabet size. (Left) Number of potential linear motif classes for different numbers of motif-discriminating positions, as a function of alphabet size. Black: 0 positions. White: 1 position. Red: 2 positions. Blue: 3 positions. Green: 4 positions. The dashed vertical line highlights the results for an alphabet size of 33 amino acids. (Right) Quotient of the number of potential linear motif classes for alphabet sizes of 33 and 20, as a function of the number of motif-discriminating positions.

Fig 5A shows the median number of potential linear motif classes as a function of alphabet size for values of *k* between 0 and 4 motif-discriminating positions. Increasing the alphabet size from 20 to 40 increases the number of potential linear motif classes in all cases. S2 Fig in S1 Data shows that this increase is highest for 0 motif-discriminating positions, decreases for values of *k* between 1 and 4 and becomes negligible for 5 or more motif-discriminating positions. When we consider an effective alphabet size of 33 amino acids (Fig 5B), the increase in the number of potential linear motif classes is more than 8-fold for 0 motif-discriminating positions, more than 4-fold for 1 motif-discriminating position and 3-fold or less for 2 or more motif-discriminating positions. In sum, increasing alphabet size on the range suggested by our knowledge of protein post-translational modifications in linear motifs increases the number of potential linear motif classes when the number of motif-discriminating positions ranges from 0 to 4. The effect of increasing alphabet size is largest when the specificity level required is zero or one motif-discriminating positions. This is notable since as we showed, a single motif-discriminating position is the norm in naturally occurring motifs.

### Sequence space occupancy

In this section, we consider the number of potential linear motif classes in the context of sequence space occupancy. A linear motif class of length *n* is a subset of a sequence space comprised of all possible 20^n^ protein subsequences. The number of potential unique instances per linear motif class is shown in Fig 1C, black bars. Half of linear motif classes contain at least 600 potential unique instances.

In the case of zero motif discriminating positions, each linear motif instance may belong to multiple classes and we were not able to find a formula for the potential occupancy of sequence space. For values of *k* of one or more motif-discriminating positions, linear motif instances belong to a single linear motif class and the potential occupancy of sequence space is simply:

$$\begin{array}{c} PotentialOccupancy\left(e,k\right)≔\prod_{1\leq i\leq n}\left(e_{i}/20\right)*\left|M\left(k\right)\right|\;\;\text{for}\;\;k>0, \end{array}$$ (10)

where the first term defines the fraction of the sequence space occupied by a linear motif class of structure **e**: = (*e*_1_, …, *e*_*n*_) ∈ {1, …, 20}^*n*^.

#### Example: [LI].C.[DE]

Let us first calculate the occupancy of sequence space for the *Rb pocket B binding ligand* linear motif class, described by the regular expression [LI].C.[DE]. This is a class of length 5, the first position admits 2 amino acids, the second any of the possible 20, the third only 1, the fourth again allows for any of the possible 20 and the fifth only allows for 2. The corresponding structure for this class is (2,20,1,20,2). The product of the permitted amino acids per position shows how many instances could belong to any single class of this structure, that is 2 * 20 * 1 * 20 * 2 = 1600. On the other hand, for a length of 5 all possible protein subsequences are 20^5^ = 3200000. The occupancy for this motif class then is the ratio between both, 1600/3200000 = 0.0005.

Next, we can apply Eq (10) to calculate the total occupancy of sequence space for all possible classes with structure (2,20,1,20,2). That is, what fraction of the sequence space would all the instances of all the possible classes occupy, for a given structure **e** and *k*. For this motif structure, the total number of possible classes is given by the second term in Eq (10). In the case of one motif discriminating position, we can substitute it by Eq (7). For the structure (2,20,1,20,2), the total number of classes is 2000 and the total occupancy of sequence space is 2000 * 0.0005 = 1. Note that we can perform the calculation in this relatively intuitive way only for *k* = 1 or higher.

#### Global results

We used the motif structures reported in the ELM database (Fig 1) and the corresponding maximum numbers of linear motif classes calculated here (Fig 4A) to estimate the potential occupancy of sequence space for values of *k* between 1 and 8. As expected from the heterogeneity in motif lengths and structures (Fig 1), the calculated values span several orders of magnitude. As done above for the number of potential motif classes, we report the median of the distribution, i.e., the median potential occupancy of sequence spaces.

**Fig 6:**
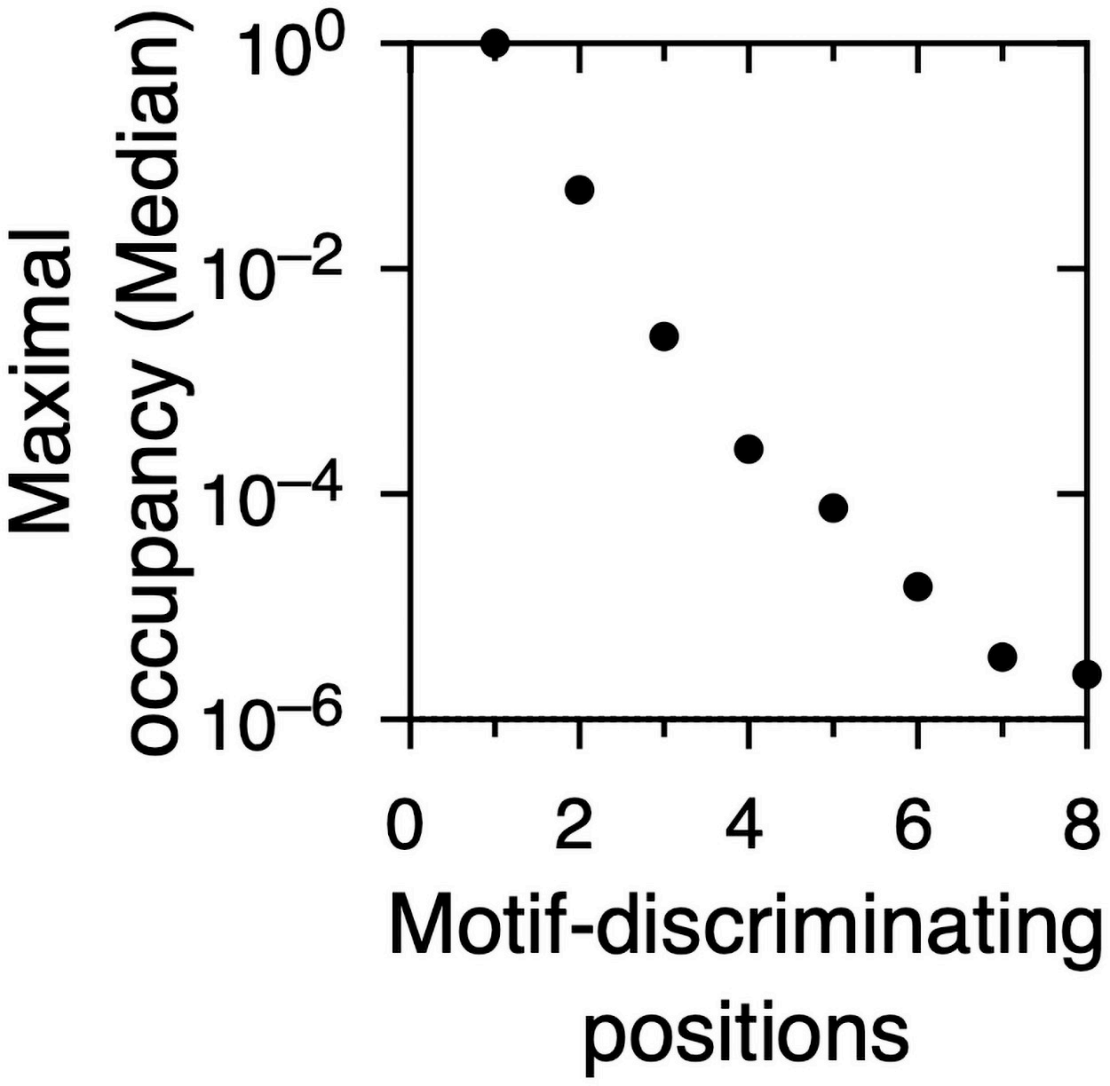
Maximal occupancy of the protein sequence space by linear motif classes as a function of the number of motif-discriminating positions and protein alphabet size. Maximal occupancy of the protein sequence space for different numbers of motif-discriminating positions.

The results are shown in S3 Fig in S1 Data and Fig 6. Curves in S3A Fig in S1 Data correspond to values of *k* from 1 to 8. As shown in 6, for *k* = 1, the potential occupancy of sequence space is 100% in all cases. For *k* = 2, the potential occupancy of sequence space is 0.05. The potential occupancy of sequence space drops steeply for values of *k* of 2 and higher. Comparison of S3A Fig in S1 Data and Fig 6 shows that the most common numbers of motif-discriminating positions maximizes the potential occupancy of sequence space by the resulting linear motif classes. For a single motif-discriminating position, all possible protein subsequences belong to a potential linear motif class.

S3B Fig in S1 Data shows the effect of increasing alphabet size on the potential occupancy of sequence space, for values of *k* from 1 to 4. For *k* = 1, the potential occupancy of sequence space is 100% regardless of alphabet size. For *k* = 2 and higher, the potential occupancy of sequence space decreases as alphabet size increases. The size of the effect is 1.7-fold for *k* = 2, 2.7-fold for *k* = 3 and 4.5-fold for *k* = 4. Upon increasing alphabet size for *k* ≥ 2, we observe a trade-off between an increasing number of potential linear motif classes (Fig 5A) and a decreasing potential occupancy of sequence space (Fig 6).

## 4 Discussion

*Are our results affected by biases in the ELM database and the use of regular expressions?* Our results may be affected by several caveats. The first two are database incompleteness and biased motif specificity. The ELM database is an incomplete sample of the existing motif classes. Moreover, it is mainly a compilation of results from low-throughput experiments driven by the biological role of specific proteins, which may bias the database towards a certain range of motif class specificities. A third caveat is related to the use of regular expressions to describe the specificity of a motif class. It is known that some motif classes present some degree of mismatch tolerance in certain positions, i.e., some motif instances that do not completely match the regular expression are functional in the cell [1]. We have used the available information to study these three issues (S4 Fig in S1 Data). We first assessed the effect of database incompleteness in our results by building ten subsampled databases sampling 25% of the motif classes in our database and recalculating the number of potential linear motif classes for 0 to 8 motif discriminating positions (S4 Fig in S1 Data, panel A). The subsampled databases overestimate the number of potential linear motif classes compared to the complete database up to two-fold. Second, we considered the effect of biased motif specificity. We sorted the motifs by the number of potential instances. This is a way of measuring motif class specificity, with more specific motif classes having a lower number of potential instances. We then split our database in two by separating the upper and lower halves of our sorted list. We recalculated the number of potential linear motif classes for each subsampled database for 0 to 8 motif discriminating positions (S4 Fig in S1 Data, panel B). On one hand, the number of potential motif classes calculated from those that are more specific than the average is within the same order of magnitude as the calculation using the full database, except for 0 discriminating positions where it is an order of magnitude lower. On the other hand, the number of potential motif classes calculated from those that are less specific than the average is within the same order of magnitude as the calculation using the full database, except for 0 discriminating positions where it is an order of magnitude higher. Last, we simulated the effect of mismatch tolerance by building two additional databases that allow all 20 amino acids at a randomly chosen position of 50% and 100% of motifs in our database. We recalculated the number of potential linear motif classes for each new database for 0 to 8 motif discriminating positions (S4 Fig in S1 Data, panel C). Tolerating a mismatch in 50% and 100% of linear motif classes in our database decreases the number of potential linear motif classes compared to the complete database around two-and four-fold respectively. From these three experiments, we interpret that database incompleteness, biased specificity and mismatch tolerance do not impact our order-of-magnitude conclusions that (1) the mild specificity of known linear motif classes maximizes the potential number of coexisting motif classes and (2) thousands of linear motif classes may remain undiscovered.

*Are regular expressions a good representation of linear motifs?* The aim of this work was to characterize how linear motif classes make use of the sequence space. We choose to describe linear motif classes in terms of regular expressions. This implies that protein subsequences not belonging to a linear motif class present at least one mismatch with the corresponding regular expression (motif versus non-motif discrimination). Our measurements of the distance in sequence space between pairs of known linear motifs (Fig 3) indicate that, in most cases, linear motif instances of a given class present at least one mismatch with the regular expression of any other class (cross-motif discrimination) (Fig 4A). We conclude that our model for the specificity of linear motif classes is in agreement with current practices in the field. The use of regular expressions allowed us to find analytical formulas for the number of potential motif classes and for sequence space occupancy, given a motif regular expression and alphabet size. These formulas may also be useful to analyze motifs in nucleic acid sequences [18].

*The specificity of linear motif classes is low, which maximizes potential motif diversity*. Our results give a general view of how biological specificity requirements shape usage of sequence space by linear motif classes. 96% of linear motif class pairs are separated in sequence space to some degree and 80% are separated by at least a single motif-discriminating position (Fig 4A). This suggests that, as some authors propose, while protein localization in time and space is relevant to determine protein-protein interactions mediated by linear motifs [7], sequence specificity can also play a significant role. The consequences of this relatively low, yet significant level of linear motif sequence specificity are remarkable: First, the observed level of specificity maximizes the number of potential linear motif classes that can coexist in a given proteome (Fig 4, panels B and C). Since the mild specificity of linear motif classes increases mutational robustness by allowing some variants to be nearly neutral in fitness terms, this in turn may play a role in organism evolvability [19]. The evolvability can be better understood by considering that nearly neutral variants might eventually lead to exaptation and the origin of new protein functionality [20].

Second, low specificity maximizes the potential occupancy of sequence space, to the point that if all potential linear motif classes are realized, all possible protein subsequences are linear motif instances (Fig 6). Since disordered protein regions have biological roles beyond harboring linear motifs, this extreme scenario seems unlikely. Refined models for coding of linear motifs may include restrictions in sequence space given by proteome size and composition in addition to linear motif specificity. The third consequence of the low specificity of linear motif classes pertains to the role of protein post-translational modifications in linear motif diversity. Our two empirical estimations based on the number of post translational modifications lead to an alphabet size close to 30 residues, significantly larger than the 20 amino acids that are usually considered. Increasing protein alphabet size in this range leads to a significant expansion in the number of potential linear motif classes (Fig 5). However, this leads in most cases to a reduction in sequence space occupancy (S3 Fig in S1 Data). The expansion of the potential linear motif repertoire is maximal and comes at a minimal cost in terms of sequence space occupancy when the specificity level at which the system operates is a single motif-discriminating position per motif pair. Altogether, we propose that the relatively low level of specificity at which known linear motif repertoires operate maximizes potential motif diversity, sequence space occupancy and the expanding effect of amino acid post-translational modifications.

*Linear motif regular expressions allow either a few or most amino acids at a given sequence position, which increases potential motif diversity*. For an alphabet size of 20 and a single motif-discriminating position, we calculate that there is a 50% chance that the number of potential linear motif classes is at least 8000 (Fig 4). We may ask how much this number depends on the highly asymmetric distribution of allowed amino acids at a motif position. For example, the most common motif structure in the database is [1, 1, 2, 20], which corresponds to 4000 potential linear motif classes separated by a single motif-discriminating position. If we assign the average number of allowed amino acids (Fig 1) at all motif positions, we obtain the structure [8, 8, 8, 8]. This structure corresponds to at most 39 potential linear motif classes separated by a single motif-discriminating position, two orders of magnitude smaller than for naturally observed linear motif structures. One classical explanation of why some positions of a motif are more constrained than others suggests that residues in the functional interface are more conserved than those that are not in the interface. For example, in the case of linear motifs binding SH3 domains [11], in the bound state some residues face the SH3 domain and are constrained to be proline, while others face the solvent and thus can be any residue. In conclusion, the structures of complexes between linear motifs and globular domains impose crucial limits to motif diversification. Since motif structure is a reduced representation of the sequence instances that allow formation of the complex between a globular protein domain and a linear motif [11], this underlines the crucial role of molecular biophysics in framing what may or may not take place at a cellular and organism scale.

*How many existing linear motif classes await discovery?* We would like to compare our calculation for the maximal number of possible motif classes with the available evidence on natural linear motifs. For the sake of simplicity, we focus on the specificity level of a single motif-discriminating position, which is fulfilled by 80% of known motif pairs (Fig 4A). In this case, we calculate 8000 potential linear motif classes for an alphabet of 20 amino acids and 36000 potential linear motif classes for an alphabet of 33 amino acids that takes into account protein post-translational modifications (Table 2). In contrast, the ELM database contains close to 300 well-characterized linear motif classes and over 3500 instances [3] (Table 2). These two figures should be regarded as lower limits because ELM is not an exhaustive database [3]. The average number of linear motif instances per linear motif class in the ELM database is 12 (Table 2, Fig 1C). In all, we predict that the potential linear motif classes outnumber the known ones by one to two orders of magnitude.

**Table 2:** Number of motif and instances from different sources. (a) Manually curated linear motif classes in the ELM database [3]. (b) Calculated from (f) and (h). (c) This work, Fig 4. (d) This work, Fig 5. (e) Manually curated linear motif instances in the ELM database [3]. (g) Calculated from (a) and (e). (f) Estimated using the ANCHOR algorithm for sequence-insensitive motif detection [9]. (h) Estimated by performing sequence searches using regular expressions and applying empirical filters to the results [21].

Linear motif classes				Linear motif instances		Average instances per linear motif class	
Known	Predicted, H. sapiens	Potential, organism-independent (this work)		Known	Predicted, H. sapiens	Known	Predicted, H. sapiens
ELM	ANCHOR & ELM regular expressions	Alphabet size 20 (c)	Alphabet size 33	ELM	ANCHOR	ELM	ELM regular expressions
289(a)	1760(b)	8000(c)	36000(d)	3523(e)	396000(f)	12(g)	225(h)

Another interesting question is how many of the potential linear motifs predicted by our model are present in a given organism. Although a quantitative answer is out of the scope of this work, we can use previous results to do a preliminary order-of-magnitude calculation for *Homo sapiens* as follows. Performing sequence searches using regular expressions and applying empirical filters to the results gives an empirical estimate of 225 linear motif instances per known linear motif class in the human proteome ([21], Table 2). Sequence-insensitive motif detection predicts that nearly two million amino acids in the human proteome belong to linear motif instances [9]. Assuming a typical motif length of 5 residues (Fig 1A) and non-overlapping motifs, we estimate 396000 linear motif instances in the human proteome. Taking 225 linear motif instances per known linear motif class in the human proteome, we calculate that there are around 396000/225 = 1760 linear motif classes in the human proteome (Table 2). Thus, the number of functional linear motif classes actually present in the human proteome might be one order of magnitude lower than the number of the possible linear motif classes. In any case, the absence of potential linear motifs in a proteome may be due to biophysical constraints not accounted for in our model and/or a fitness landscape that led to an incomplete exploration of the linear motif space, which is a relatively recent evolutionary innovation [5].

Altogether, the figures shown on Table 2 suggest that not all possible linear motif classes and instances are realized in all organisms and that we are only beginning to describe those that exist in nature.

*Which linear motif classes are likely to be discovered in the future?* Hundreds of linear motif classes may be present in the human proteome, awaiting discovery. It is interesting to consider this dark matter of linear motif diversity [22] from the viewpoint of hidden heterogeneity in the the globular domains that form complexes with linear motifs. For example, over a hundred of related, yet different SH3 domains are present in the human proteome. The first of these domains to be characterized were reported to bind linear motifs described by the RxxPxxP (+) and PxxPxxR (-) regular expressions [11]. However, it was later reported that SH3 domains are functionally diverse in that some of them do not interact with the linear motifs described above [11]. This suggests that some of the globular domain families currently associated with a single linear motif class may be associated in the future with multiple linear motif classes [23]. Another likely source of hidden linear motif diversity are domains of unknown function: Several thousands of globular domains lack a known molecular activity and may function through their interaction with currently uncharacterized linear motif classes [24].

The discovery of linear motifs resembles the species discovery curve in ecology, i.e., the cumulative number of species recorded in a site as a function of the surveyed area [8]. The shape of a discovery curve depends critically on both the relative abundances of species and the sampling methods used [8]. A comprehensive characterization of the linear motifs in a natural proteome may require a quantitative study of the commonness and rarity of individual motifs and a combination of high- and low-throughput sampling methods able to detect motifs of low abundance.

## Supporting information

S1 Data (PDF)
