# Supplementary material for: Thousands of protein linear motif classes may still be undiscovered
Source: PLoS One. 2021 May 3;16(5):e0248841. doi: 10.1371/journal.pone.0248841 (PMC8092775; doi:10.1371/journal.pone.0248841)
Supplement: S1 Data — (PDF) [file pone.0248841.s001.pdf]

S1 Data

S1 File. Database of linear motif classes and regular expressions.

CLV_C14_Caspase37 [DSTE][^P][^DEWHFYC]D[GSAN]

CLV_MEL_PAP_1 [ILV]..R[VF][GS].

CLV_NRD_NRD_1 (.RK)|(RR[^KR])

CLV_PCSK_FUR_1 R.[RK]R.

CLV_Separin_Fungi S[IVLMH]E[IVPFMLYAQR]GR.

CLV_Separin_Metazoa E[IMPVL][MLVP]R.

CLV_TASPASE1 Q[MLVI]DG..[DE]

DEG_APCC_DBOX_1 .R..L..[LIVM].

DEG_APCC_KENBOX_2 .KEN.

DEG_APCC_TPR_1 .[ILM]R$

DEG_COP1 [DE][DE]...VP[DE]

DEG_CRL4_CDT2_1 [NQ]{0,1}..[ILMV][ST][DEN][FY][FY].{2,3}[KR]{2,3}[^DE]

DEG_MDM2_1 F...W..[LIV]

DEG_Nend_UBRbox_4 ^M{0,1}(C).

DEG_ODPH_VHL_1 [IL]A(P).{6,8}[FLIVM].[FLIVM]

DEG_SCF_COI1_1 ..[RK][RK].SL..F[FLM].[RK]R[HRK].[RK].

DEG_SCF_FBW7_1 [LIVMP].{0,2}(T)P..([ST])

DEG_SCF_SKP2-CKS1_1 ..[DE].(T)P.K

DEG_SCF_TIR1_1 .[VLIA][VLI]GWPP[VLI]...R.

DEG_SCF_TRCP1_1 D(S)G.{2,3}([ST])

DEG_SIAH_1 .P.A.V.P[^P]

DOC_AGCK_PIF_1 F..[FWY][ST][FY]

DOC_ANK_TNKS_1 .R..[PGAV][DEIP]G.

DOC_CKS1_1 [MPVLIFWYQ].(T)P..

DOC_CYCLIN_1 [RK].L.{0,1}[FYLIVMP]

DOC_MAPK_1 [KR]{0,2}[KR].{0,2}[KR].{2,4}[ILVM].[ILVF]

DOC_MAPK_2 F.FP

DOC_PIKK_1 [DEN][DEN].{2,3}[ILMVA][DEN][DEN]L

DOC_PP1_RVXF_1 ..[RK].{0,1}[VIL][^P][FW].

DOC_PP1_SILK_1 .[GS]IL[KR][^DE]

DOC_PP2B_1 .P[^P]I[^P][IV][^P]

DOC_PP2B_2 L.[LIVAPM]P

DOC_SPAK_OSR1_1 RF[^P][IV].

DOC_USP7_1 [PA][^P][^FYWIL]S[^P]

DOC_USP7_2 P.E[^P].S[^P]

DOC_WD40_RPTOR_TOS_1 F[EDQS][MILV][ED][MILV]((.{0,1}[ED])|($))

DOC_WW_Pin1_4 ...([ST])P.

LIG_14-3-3_1 R.[^P]([ST])[^P]P

LIG_Actin_RPEL_3 [IL]..[^P][^P][^P][^P]R.....[IL]..[^P][^P][ILV][ILM]

LIG_Actin_WH2_2 [^R]..((.[ILMVF])|([ILMVF].))[^P][^P][ILVM].{4,7}L(([KR].)|(NK))[VATIGS]

LIG_AP2alpha_1 F.D.F

LIG_AP2alpha_2 DP[FW]

LIG_APCC_Cbox_2 DR[YFH][ILFVM][PA]..

LIG_AP_GAE_1 [DE][DES][DEGAS]F[SGAD][DEAP][LVIMFD]

LIG_BIR_III_3 ^M{0,1}A.[AP].

LIG_BRCT_BRCA1_1 .(S)..F

LIG_CaMK_CASK_1 ((SP)|([ED].{0,1}))[IV]W[IVL].R

LIG_CAP-Gly_1 [ED].{0,2}[ED].{0,2}[EDQ].{0,1}[YF]$

LIG_CAP-Gly_2 .W[RK][DE]GCY$

LIG_Clathr_ClatBox_1 L[IVLMF].[IVLMF][DE]

LIG_Clathr_ClatBox_2 .[NP]W[DES].W

LIG_CORNRBOX L[^P]{2,2}[HI]I[^P]{2,2}[IAV][IL]

LIG_CtBP_PxDLS_1 (P[LVIPME][DENS][LM][VASTRG])|(G[LVIPME][DENS][LM][VASTRG]((K)|(.[KR])))

LIG_Dynein_DLC8_1 [^P].[KR].TQT

LIG_EABR_CEP55_1 .A.GPP.{2,3}Y.

LIG_EF_ALG2_ABM_1 P[PG]{0,1}YP.{1,6}Y[QS]{0,1}P

LIG_EH_1 .NPF.

LIG_EH1_1 .[FYH].[IVM][^WFYP][^WFYP][ILM][ILMV].

LIG_eIF4E_1 Y....L[VILMF]

LIG_EVH1_1 ([FYWL]P.PP)|([FYWL]PP[ALIVTFY]P)

LIG_EVH1_2 PP..F

LIG_EVH1_3 [FY].[FW].....[LMVIF]P.P[DE]

LIG_FAT_LD_1 [LV][DE][^P][LM][LM][^P][^P]L[^P]

LIG_FHA_1 ..(T)..[ILV].

LIG_FHA_2 ..(T)..[DE].

LIG_GLEBS_BUB3_1 [EN][FYLW][NSQ].EE[ILMVF][^P][LIVMFA]

LIG_GYF [QHR].{0,1}P[PL]PP[GS]H[RH]

LIG_HCF-1_HBM_1 [DE]H.Y

LIG_HOMEOBOX [FY][DEP]WM

LIG_HP1_1 P[MVLIRWY]V[MVLIAS][LM]

LIG_Integrin_isoDGR_1 NGR

LIG_IQ ...[SACLIVTM]..[ILVMFCT]Q.{3,3}[RK].{4,5}[RKQ]..

LIG_KEPE_2 [VILMFT]K.EP.{2,3}[DE]

LIG_LIR_Gen_1 [EDST].{0,2}[WFY]..[ILV]

LIG_LYPXL_S_1 [LM]YP.[LI]

LIG_MAD2 [KR][IV][LV].....P

LIG_MYND_1 P.L.P

LIG_MYND_2 PP.LI

LIG_MYND_3 [LMV]P.LE

LIG_NBox_RRM_1 F..A[ILV]..A..[ILV]

LIG_NRBOX [^P]L[^P][^P]LL[^P]

LIG_OCRL_FandH_1 .F[^P][^P][KRIL]H[^P][^P][YLMFH][^P]...

LIG_PAM2_1 ..[LFP][NS][PIVTAFL].A..(([FY].[PYLF])|(W..)).

LIG_PAM2_2 ((WPP)|([FL][PV][APQ]))EF.PG.PWKG.

LIG_PDZ_Class_1 ...[ST].[ACVILF]$

LIG_PDZ_Class_2 ...[VLIFY].[ACVILF]$

LIG_PDZ_Class_3 ...[DE].[ACVILF]$

LIG_PTAP_UEV_1 .P[TS]AP.

LIG_PTB_Apo_2 (.[^P].NP.[FY].)|(.[ILVMFY].N..[FY].)

LIG_Rb_LxCxE_1 [LI].C.[DE]

LIG_Rb_pABgroove_1 ..[LIMV]..[LM][FY]D.

LIG_RGD RGD

LIG_RRM_PRI_1 .[ILVM]LG..P.

LIG_SH2_GRB2 (Y).N.

LIG_SH2_PTP2 (Y)[IV].[VILP]

LIG_SH2_SRC (Y)[QDEVAIL][DENPYHI][IPVGAHS]

LIG_SH2_STAT3 (Y)..Q

LIG_SH2_STAT5 (Y)[VLTFIC]..

LIG_SH2_STAT6 G(Y)[KQ].F

LIG_SH3_1 [RKY]..P..P

LIG_SH3_2 P..P.[KR]

LIG_SH3_3 ...[PV]..P

LIG_SH3_4 KP..[QK]...

LIG_SH3_5 P..DY

LIG_Sin3_1 [LIV]..[LM]L.AA.[FY][LI]

LIG_Sin3_2 [FHYM].A[AV].[VAC]L[MV].[MI]

LIG_Sin3_3 [FA].[LA][LV][LVI]..[AM]

LIG_SPRY_1 [ED][LIV]NNN[^P]

LIG_SUFU_1 [SV][CY]GH[LIF][LAST][GAIV].

LIG_SUMO_SBM_1 [ILV](.[ILV]|[ILV]|[ILV].)[ILV][STDE]{1,10}

LIG_SUMO_SBM_2 [STDE]{1,10}[ILV](.[ILV]|[ILV]|[ILV].)[ILV]

LIG_SxIP_EBH_1 ([KR][^ED]{0,5}[ST].IP[^ED]{5,5})|([^ED]{5,5}[ST].IP[^ED]{0,5}[KR])

LIG_TPR EEVD$

LIG_TRAF2_1 [PSAT].[QE]E

LIG_TRAF2_2 P.Q..D

LIG_TRAF6 ..P.E..[FYWHDE].

LIG_TRFH_1 [FY].L.P

LIG_TYR_ITAM [DEN]..(Y)..[LI].{6,12}(Y)..[LI]

LIG_TYR_ITIM [ILV].(Y)..[ILV]

LIG_TYR_ITSM ..T.(Y)..[IV]

LIG_ULM_U2AF65_1 [KR]{1,4}[KR].[KR]W.

LIG_WD40_WDR5_1 [ED].{0,3}[VI]D[VI]

LIG_WD40_WDR5_WIN_1 [HN].[HNST]G[SCA]AR[STAC][EQ][GPVILM][YFHKRQN][YHLIVMATS]

LIG_WH1 ES[RK][FY].F[HR][PST][IVLM][DES][DE]

LIG_WRPW_1 [WFY]RP[WFY].{0,7}$

LIG_WW_1 PP.Y

LIG_WW_2 PPLP

LIG_WW_3 .PPR.

MOD_ASX_betaOH_EGF C.([DN]).{4,4}[FY].C.C

MOD_CAAXbox (C)[^DENQ][LIVM].$

MOD_CDK_1 ...([ST])P.[KR]

MOD_CK1_1 S..([ST])...

MOD_CK2_1 ...([ST])..E

MOD_CMANNOS (W)..W

MOD_GlcNHglycan [ED]{0,3}.(S)[GA].

MOD_GSK3_1 ...([ST])...[ST]

MOD_LATS_1 H.[KR]..([ST])[^P]

MOD_NEK2_1 [FLM][^P][^P]([ST])[^DEP][^DE]

MOD_N-GLC_1 .(N)[^P][ST]..

MOD_N-GLC_2 (N)[^P]C

MOD_NMyristoyl ^M{0,1}(G)[^EDRKHPFYW]..[STAGCN][^P]

MOD_OFUCOSY C.{3,5}([ST])C

MOD_OGLYCOS C.(S).PC

MOD_PIKK_1 ...([ST])Q..

MOD_PK_1 [RK]..(S)[VI]..

MOD_PKA_1 [RK][RK].([ST])[^P]..

MOD_PKB_1 R.R..([ST])[^P]..

MOD_PLK .[DE].([ST])[ILFWMVA]..

MOD_ProDKin_1 ...([ST])P..

MOD_SPalmitoyl_2 G(C)M[GS][CL][KP]C

MOD_SPalmitoyl_4 ^M{0,1}G(C)..S[AKS]

MOD_SUMO [VILMAFP](K).E

MOD_TYR_CSK [TAD][EA].Q(Y)[QE].[GQA][PEDLS]

MOD_TYR_DYR ..[RKTC][IVL]Y[TQHS](Y)[IL]QSR

MOD_WntLipid [ETA](C)[QERK]..F...RWNC[ST]

TRG_AP2beta_CARGO_1 [DE].{1,2}F[^P][^P][FL][^P][^P][^P]R

TRG_Cilium_RVxP_2 RV.P.

TRG_ENDOCYTIC_2 Y..[LMVIF]

TRG_ER_diArg_1 ([LIVMFYWPR]R[^YFWDE]{0,1}R)|(R[^YFWDE]{0,1}R[LIVMFYWPR])

TRG_ER_diLys_1 K.{0,1}K.{2,3}$

TRG_ER_FFAT_1 [DE].{0,4}E[FY][FYK]D[AC].[ESTD]

TRG_ER_KDEL_1 [KRHQSAP][DENQT]EL$

TRG_Golgi_diPhe_1 Q.{6,6}FF.{6,7}$

TRG_LysEnd_APsAcLL_1 [DERQ]...L[LVI]

TRG_LysEnd_GGAAcLL_1 D..LL.{1,2}$

TRG_LysEnd_GGAAcLL_2 S[LW]LD[DE]EL[LM]

TRG_NES_CRM1_1 ([DEQ].{0,1}[LIM].{2,3}[LIVMF][^P]{2,3}[LMVF].[LMIV].{0,3}[DE])|([DE].{0,1}[LIM].{2,3}[LIVMF][^P]{2,3}[LMVF].[LMIV].{0,3}[DEQ])

TRG_NLS_MonoCore_2 [^DE]((K[RK])|(RK))[KRP][KR][^DE]

TRG_PEX_1 W...[FY]

TRG_PEX_2 F...[WF]

TRG_PEX_3 L..LL...L..F

TRG_PTS1 (.[SAPTC][KRH][LMFI]$)|([KRH][SAPTC][NTS][LMFI]$)

S1 Table. Redundant motifs that where discarded and the motif that represents the group.

Discarded motifs	Representative motif
DEG_CRL4_CDT2_2	DEG_CRL4_CDT2_1
DEG_SCF_FBW7_2	DEG_SCF_FBW7_1
DOC_AGCK_PIF_2 and DOC_AGCK_PIF_3	DOC_AGCK_PIF_1
LIG_14-3-3_2 and LIG_14-3-3_3	LIG_14-3-3_1
LIG_Actin_WH2_1	LIG_Actin_WH2_2
LIG_APCC_Cbox_1	LIG_APCC_Cbox_2
LIG_BIR_III_1, LIG_BIR_III_2 and LIG_BIR_III_4	LIG_BIR_III_3
LIG_BRCT_BRCA1_2 and LIG_BRCT_MDC1_1	LIG_BRCT_BRCA1_1
LIG_EF_ALG2_ABM_2	LIG_EF_ALG2_ABM_1
LIG_eIF4E_2	LIG_eIF4E_1
LIG_KEPE_1 and LIG_KEPE_3	LIG_KEPE_2
LIG_LIR_Apic_2, LIG_LIR_LC3C_4 and LIG_LIR_Nem_3	LIG_LIR_Gen_1
LIG_LYPXL_L_2	LIG_LYPXL_S_1
LIG_PTB_Phospho_1	LIG_PTB_Apo_2
LIG_WD40_WDR5_2	LIG_WD40_WDR5_1
LIG_WD40_WDR5_WIN_2 and LIG_WD40_WDR5_WIN_3	LIG_WD40_WDR5_WIN_1
LIG_WRPW_2	LIG_WRPW_1
MOD_PKA_2	MOD_PKA_1
TRG_Cilium_Arf4_1	TRG_Cilium_RVxP_2
TRG_LysEnd_APsAcLL_3	TRG_LysEnd_APsAcLL_1
TRG_NLS_Bipartite_1, TRG_NLS_MonoExtC_3 and TRG_NLS_MonoExtN_4	TRG_NLS_MonoCore_2
CLV_PCSK_KEX2_1, CLV_PCSK_PC1ET2_1, CLV_PCSK_PC7_1 and CLV_PCSK_SKI1_1	CLV_PCSK_FUR_1

S2 Table. Distribution of post translational modifications in Uniprot database.

Category	Number of different modifications	Frequency in Uniprot DB	%
Modified residues	289	240935	48.2
Cross links	124	12374	2.5
Glycosylation sites	69	113720	22.7
Lipidations	39	12792	2.5
Disulfide bond	1	120084	24.1
Total	522	499905	100

S3 Table. Distribution of the twelve most frequent post translational modifications.

Category	Modification	Frequency in Uniprot DB	% of Uniprot
Modified residues	phosphoserines	117588	23.5
	phosphothreonines	24845	5
	N6-acetyllysines	23012	4.6
	phosphotyrosines	9403	1.9
	N6-succinyllysines	6974	1.4
	N6-(pyridoxal phosphate)lysines	6574	1.3
	N-acetylalanines	4131	0.8
	N-acetylmethionine	3346	0.7
Cross links	Glycyl lysine isopeptides	9776	1.9
Glycosylation sites	N-linked (GlcNAc...)	107946	21.6
Lipidations	S-palmytoil cysteines	3811	0.8
Disulfide bond	disulfide bonds	120084	24.1
Total	12	437490	87.6

S4 Table. Some motifs that depend on modified residues.

Modified residue	Dependent motifs
phosphotyrosine	SH2 binding, PTB binding
phosphoserine	DEG_SCF_TRCP1_1, LIG_IRF3_LxIS_1, BRCT binding,WD40
phosphothreonine	DEG_SCF_TRCP1_1, WD40 binding, Cks1-binding, FHA binding
hydroxyproline	DEG_ODPH_VHL_1
isoaspartate	LIG_Integrin_isoDGR_1
sulfinic/sulfonic acid	DEG_Nend_UBRbox_4
argynilated cysteine	DEG_Nend_UBRbox_4

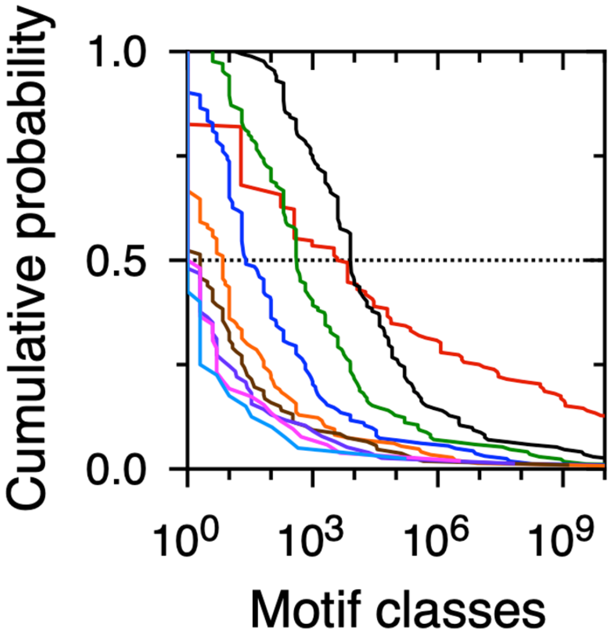

S1 Fig. Number of potential linear motif classes as deduced from the ELM database. Cumulative distribution function of the number of potential linear motif classes for different numbers of motif-discriminating positions. Red: 0 positions. Black: 1 position. Green: 2 positions. Blue: 3 positions. Orange: 4 positions. Brown: 5 positions. Purple: 6 positions. Pink: 7 positions. Cyan: 8 positions.

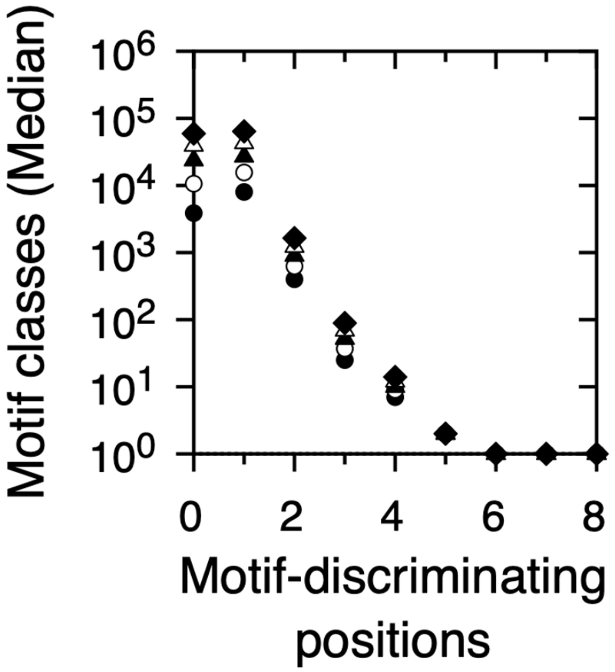

S2 Fig. Number of potential linear motif classes as a function of protein alphabet size. Number of potential linear motif classes for different alphabet sizes, as a function of the number of motif-discriminating positions. Black circle: 20 amino acids. Empty circle: 25 amino acids. Black triangle: 30 amino acids. Empty triangle: 35 amino acids. Black diamond: 40 amino acids.

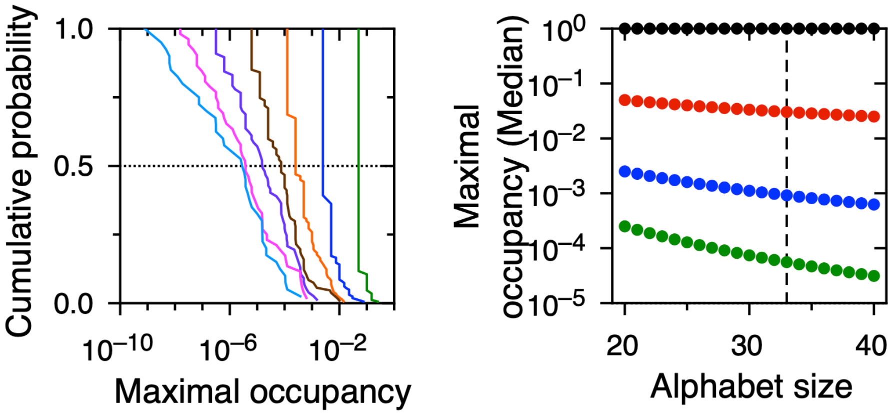

S3 Fig. Maximal occupancy of the protein sequence space by linear motif classes as a function of the number of motif-discriminating positions and protein alphabet size. (Left) Cumulative distribution function of the maximal occupancy of the protein sequence space for different numbers of motif-discriminating positions. Green: 2 positions. Blue: 3 positions. Orange: 4 positions. Brown: 5 positions. Purple: 6 positions. Pink: 7 positions. Cyan: 8 positions. Maximal occupancy for 1 motif-discriminating positions is 1 in all cases. (Right) Maximal occupancy of the protein sequence space for different numbers of motif-discriminating positions, as a function of alphabet size. The dashed vertical line highlights the results for an alphabet size of 33 amino acids. Black: 1 position. Red: 2 positions. Blue: 3 positions. Green: 4 positions.

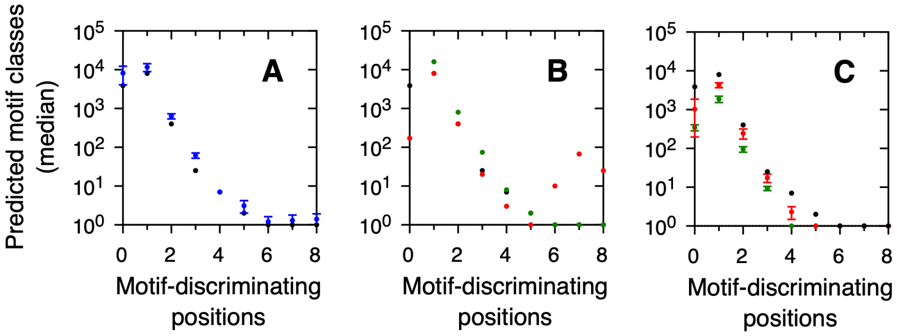

S4 Fig. Effect of database incompleteness, biased specificity and mismatch tolerance on the number of potential linear motif classes as deduced from the ELM database. The number of potential linear motif classes for different numbers of motif-discriminating positions for the full database is plotted in all panels as a reference (black circles). (A) Number of potential linear motif classes for different numbers of motif-discriminating positions for ten databases sampling 25% of the motif classes in our database (blue circles). (B) Number of potential linear motif classes for different numbers of motif-discriminating positions for motifs having a higher number of potential instances than the average (i.e., lower specificity) (green circles) and for motifs having a lower number of potential instances than the average (i.e., higher specificity) (red circles). (C) Number of potential linear motif classes for different numbers of motif-discriminating positions for two modified databases that allow all 20 amino acids at a randomly chosen position of 50% and 100% of motifs in our database (red and green circles, respectively).
